# Motherhood Health Penalty: Impact of Fertility on Physical and Mental Health of Chinese Women of Childbearing Age

**DOI:** 10.3389/fpubh.2022.787844

**Published:** 2022-05-20

**Authors:** Yao Jiang, Fan Yang

**Affiliations:** ^1^Zhou Enlai School of Government, Nankai University, Tianjin, China; ^2^Department of Labor and Social Security, School of Public Administration, Sichuan University, Chengdu, China

**Keywords:** motherhood, health of women, women healthcare, gender role, health policy

## Abstract

**Background:**

The negative consequences of childbearing on mothers are called the motherhood penalty, and it manifests in the aspects of women's physical and mental health. In May 2021, China relaxed its birth policy that allowed a married couple to have three children. It gives women the opportunity to have more children, but also may increase more risks to mothers' physical and mental health.

**Objectives:**

The objectives of this study were to clarify the relationships between the fertility and the physical/mental health of women of childbearing age and empirically confirm the existence of the motherhood health penalty in China.

**Materials and Methods:**

Using a nationally representative dataset from the China Labor-force Dynamics Survey 2018, we examined the effects of fertility on the physical and mental health of Chinese women of childbearing age. Physical health was self-rated, and mental health was assessed according to the Center for Epidemiological Studies Depression scale. The instrumental variable approach and the models of inverse probability of treatment weighting of propensity scores and regression adjustment were employed to overcome the endogeneity between fertility and health of women.

**Results:**

The empirical results showed that the total number of births had significant adverse impacts on the physical and mental health of women of childbearing age, which empirically demonstrated the existence of the motherhood health penalty in China. The results of heterogeneity analysis indicated that the physical and mental health of the rural women was more easily affected by childbearing compared with that of the urban samples. In a mechanism analysis, the pathways of income and the multiple roles played by mothers were found to mediate the impacts of the total number of births on the physical and mental health of women. The robustness checks showed that the results of this study were robust.

**Conclusions:**

The findings of this study extend the motherhood penalty to the health domain, and they have important implications for improving healthcare policy for women of childbearing age in China and other countries and regions and promoting gender equality in the healthcare field.

## Introduction

The negative consequences of childbearing on mothers are collectively called the motherhood penalty, which is manifested in many aspects (1–4). It is acknowledged that in many cultures, the mothers suffer a wage disadvantage compared with non-mothers in the labor market; this disparity is known as the motherhood wage penalty (5, 6). Following the prior studies on the motherhood wage penalty (1–6), we focused on the negative effects of childbearing on the physical and mental health of the mothers of childbearing age, namely, the motherhood health penalty.

**Figure 1:**
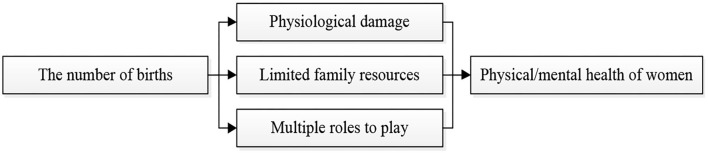
Pathways through which the fertility affects the health of women.

Although it is widely accepted that childbearing is correlated with the worse physical and mental health outcomes for women, the theoretical explanations for this phenomenon are diverse. The explanations for the negative associations between childbearing and women's health outcomes can be broadly grouped into the following three categories: The physiological damage of fertility, the limited family resources, and the multiple roles played by mothers (Figure 1).

The first theoretical explanation for the health effects of childbearing on women is physiological. From the physiological perspective, a greater number of births means the shorter interbirth intervals, which increases the possibility of physical damage, postnatal or prenatal depression, and mortality of the mother (7–9). One study demonstrated that the women who gave birth to more children were more prone to anxiety and depression symptoms (10). The one-child policy implemented in China was also proven to significantly reduce the physical damage and mortality of women by reducing the total number of pregnancies and births (11). Some evidence-based medical studies and meta-analyses have suggested that the process of childbearing and the maintenance of body functions compete for resources, and childbearing tends to cause lingering effects in the form of chronic diseases (12–14); for example, the childbearing affects mother's lipid and glucose metabolism, and the higher the number of births, the more likely the occurrences of obesity and chronic arteriosclerosis (15).

The second explanation for the motherhood health penalty is that the childbearing reduces family resources for mothers. From the perspective of family resource allocation, the economic budget in a family is constrained and limited (16). To provide the children with a better life, when family resources are limited, mothers may choose to sacrifice their own welfare; for example, decreasing their health investment and nutritional intake (17). Therefore, the limited family resources allocated to mothers are reduced, and the childbearing leads to an adverse impact on their health status. A study focusing on the impact of child structure on mothers' health found that in rural areas of China, women with hypertension who had more sons were less likely to receive medical treatment for the condition (18). This is a reflection of the family resource concessions and health sacrifices made by mothers for their children.

The third explanation for the motherhood health penalty is the multiple roles that mothers need to play. In China, the mother has been traditionally seen as the primary caregiver of a family; thus, the mothers are generally considered to be responsible for performing the housework, taking care of husbands and aged parents, and bearing and caring for children (19). Correspondingly, women play multiple roles, including wife, child, and mother. With the development of socioeconomic status, women have gradually moved beyond these family roles, attempting to shed the stereotypical gender image of the traditional housewife by becoming professionals, despite the associated occupational stress (20–23). However, according to the energy/time limitation theory, people have finite energy and time, and the multiple roles of women consume considerable amounts of both (24). A higher number of births suggests that women need to dedicate much more effort to housework and childcare, take longer career breaks, and experience more professional stress, which might be detrimental to their health (25).

Although these three explanations from the existing literature indeed link the negative effects of fertility to mothers' physical and mental health outcomes, studies demonstrating the relationship between fertility and health outcomes and the existence of the motherhood health penalty in China remain elusive. First, when discussing the negative impact of reproductive behavior on women's health, the previous studies have mainly examined the women of all ages with childbearing experience. In terms of the Chinese context considered in this study, the relaxation of the birth policy primarily affected the number of births of women of childbearing age, rather than women of all ages. Therefore, it is of practical significance to focus on women of childbearing age as the study subject. Second, a few studies have clarified the relationship between the fertility and mothers' physical and mental health from an empirical perspective due to the endogeneity caused by potential confounding factors. Third, although the pathways through which fertility impacts the physical and mental health of women were discussed in the existing literature, studies largely focused on the physiological damage aspect based on medical analysis, whereas few studies attempted to empirically examine the pathways of family resources and multiple roles.

In this study, we attempted to address these three research gaps. First, based on a nationally representative dataset from the China Labor-force Dynamics Survey 2018 (CLDS2018), we focused on women of childbearing age, which was a practical contribution of this study. The findings in this study can aid policy makers in establishing targeted health policies and supports that can improve the health of women of childbearing age in developing countries and regions. Second, to deal with the endogeneity caused by potential confounding factors, we adopted a pertinent instrumental variable (IV)—gender of the first child, and used the inverse probability of treatment weighting of propensity scores followed by regression adjustment (IPTW–RA) for managing multiple treatment groups. Third, we empirically tested the potential pathways of family resources and the multiple roles. In this way, we connected fertility with the health of women of childbearing age.

The remainder of this study is organized as follows: In section Chinese Context, we described the Chinese context of the motherhood health penalty. In section Materials and Methods, the method adopted in this study was described in detail, including data, the measurements of variables, and empirical models. In section Results, the empirical results were provided. The discussion, policy implications, and limitations of this study were discussed in section Discussion. Section Conclusions presented our conclusions.

## Chinese Context

The evolution of the birth policy, traditional cultural practices, and the improvement of women's social status all make China an ideal research background in which to explore the effects of fertility on the physical and mental health of women of childbearing age.

The birth policy evolution and traditional cultural practices of patriarchy in China provide a perspective for re-examining the health status of women of childbearing age. After the foundation of the People's Republic of China, the stable living environment led to an increase in the natural growth rate of the population, which, at one point, reached 22% (26). To control the country's explosive population growth, in 1980, China's one-child policy for married couples was formalized: In general circumstances, a married couple could only have one child. This birth quota was strictly enforced with punitive measures for violations (26). The couples who exceeded the birth quota were required to pay expensive social compensation fees, and the civil servant couples who violated the policy faced additional administrative punishment or disciplinary sanctions (27). However, for many centuries, the Chinese society pursued a patriarchal family system and preferred sons over daughters (28, 29). The traditional preference for sons and discrimination against daughters were more predominant in the underdeveloped rural areas than the urban areas (30). Therefore, in 1988, to prevent sex-selective abortion, the Chinese government amended the one-child policy and allowed some couples to have a second child under certain conditions; the main exception to the policy applied to the couples who lived in certain rural areas and whose first child was a girl (31, 32). Although the one-child policy was amended, the adjustment was only implemented in some underdeveloped rural areas, not all rural areas, let alone the urban areas. Thus, when the first child was a girl, parents who preferred a son but did not satisfy the certain conditions (i.e., couples in an urban area or in a rural area where couples were not permitted to have a second child even if the first child was a girl) were willing to take the risk of receiving strict penalties to have more children.

Despite violations of the policy, a prior study found that the one-child policy played a crucial role in controlling the population of China, preventing the addition of approximately 200 million people (33). However, in today's Chinese context, the demographic dividends are disappearing, and the fertility rate is gradually declining owing to the previously implemented strict one-child policy (34, 35). The progressive relaxation of the birth policy has been a major trend to cope with these practical issues. In 2015, the universal two-child policy (allowing a married couple to have two children) was established, which marked the end of the one-child policy after being implemented for 35 years (36). In 2021, the three-child policy was promulgated, allowing a married couple in China to have three children (37).

However, with the birth policy evolution and the cultural practices of patriarchy, childbearing age women's status of fertility subject seems to be ignored to a certain extent. Women endure the government's regulation of birth quotas and the family's expectation of sons. Therefore, because of the birth policy and the cultural practices of patriarchy, mothers who violate the birth policy bear an economic burden due to social compensation fees, as well as the health burdens associated with bearing sons.

Despite China's historical tradition of prioritizing boys over girls, women's social status has continually improved since the foundation of the People's Republic of China. In 1949, to promote gender equality in the political and work domains, the slogan “women can hold up half of the sky” was put forward, and “equal pay for equal work” was written into the Chinese Constitution (38, 39). In the sphere of private life, the Marriage Law in 1950 stipulated the freedom of marriage and explicitly stated that in the marital relationship, the rights and interests of the wife were equal to those of the husband (40). The improving social status of women enhanced their ability to attain education, narrowed the gender gap in the labor market, and decreased household inequality. With their improving social status, millions of women of childbearing age have increasingly suffered a more stressful role, “the working mother” (22). As a country strongly influenced by Confucian culture, raising offspring and continuing the family line are crucial filial responsibilities of women (41). The traditional Chinese family culture advocated that “there are three forms of unfilial conduct, of which the worst is to have no descendants.” Working mothers face a conflict between family and work, namely, the conflict between traditional values and modern ideas (23). Therefore, the Chinese women of childbearing age suffer stress from their families and workplaces, and their health becomes vulnerable.

## Materials and Methods

### Data

The data employed in this study are from the CLDS2018. The CLDS2018 was conducted by Sun Yat-sen University, and it resulted in one of the largest and most nationally representative datasets in China. The main content of the dataset included respondents' personal characteristics (e.g., birth year, education, income, and marital status) and family characteristics (e.g., the number of family members and self-rated family social status). The dataset was collected from respondents in 29 provincial administrative units of China to ensure its representativeness.

In this study, we focused on women of childbearing age, which was defined by the United Nations as 15–49 years old (42). Consequently, after excluding samples with invalid answers (inapplicable, unclear, and missing answers), 4,245 observations of women of childbearing age were used in this study.

### Measurements

#### Explained Variables

The explained variables are the physical and mental health of women of childbearing age. For physical health, self-ratings were used. Respondents were asked, “How do you evaluate your current health?” The answer was given as a number on a five-point Likert scale ranging from “1” (very unhealthy) to “5” (very healthy). Considering the subjectivity of self-rated health, to test the robustness of the results, we employed the objective index of body mass index (BMI) as the alternative explained variable. According to the World Health Organization, a normal BMI between 18.5 and 23 (18.5 ≤ BMI < 23) is appropriate for Asians (43). Therefore, in this study, the range of BMI from 18.5 to 23 (18.5 ≤ BMI <23) was regarded as normal range in physical health. If respondent's BMI ranged from 18.5 to 23 (18.5 ≤ BMI <23), the variable of BMI was coded as “1”; otherwise, “0.”

The mental health of the sampled women was assessed by using the Center for Epidemiological Studies Depression (CES-D) scale. The CES-D scale is widely used for assessing mental health, and the prior studies have confirmed its validity and reliability for mental health assessment in China (44, 45). The total score of the CES-D scale ranged from “20” to “80.” The higher the CES-D score, the deeper the depression, and the worse the mental health.

#### Explanatory Variable

The total number of births by women of childbearing age is the core explanatory variable. It was obtained by asking respondents the question, “How many children have you had?” The variable of the total number of births was numerical.

#### Mediating Variables

To explore the potential channels through which the total number of births impacts the physical and mental health of women of childbearing age, we empirically tested the mediating effects of family resource limitations and the multiple roles of women. Income is the pillar of family resources and provides vital support that allows women to maintain good health (46). Compared to the high-income women, the low-income women may face more financial restrictions on health investment and, ultimately, have the worse health outcomes (47). Therefore, in this study, we used income as the proxy variable of family resources. Income was a continuous variable and was measured by the total annual income of respondents in 2017, incorporating wage income, operating income, property income, and transfer income. Self-sufficient agricultural production was converted into total income according to market value. In the empirical analysis, we used the natural logarithm of income.

The variable of multiple roles was binary and was determined by asking two questions: “Have you ever engaged in work with income in the past year?” and “Are you responsible for the household chores (cooking, washing, and sweeping) and childcare?” The variable of multiple roles was coded “1” if the answers to the two questions were both “Yes” and was “0” otherwise.

#### Control Variables

To determine the effects of fertility on the physical and mental health of women of childbearing age, we controlled the following variables: Age, education, lifestyles (smoking, drinking, and regular exercise), Hukou, marital status, father, mother, migrant experience, and regional effect.

**Age:** Because an individual's health deteriorates with increment of age, age is a vital factor influencing an individual's physical and mental health (48). Age was a continuous variable in this study.

**Education:** People with a higher level of education tend to have better healthcare literacy and better health outcomes (49). Education was measured by years of schooling.

**Lifestyles:** Smoking, drinking, and regular exercise are all the factors that affect health (50). The respondents were asked, “Do you have the habit of smoking/drinking/regular exercise?” This variable was coded “1” for “yes” and “0” otherwise.

**Hukou:** This variable represents the area where the respondent's household is registered (“Hukou” in Chinese), that is, rural or urban household registration (51). There is a disparity in medical security development between urban and rural areas in China; medical security is closely related to the area where an individual's household is registered (52). Thus, the area where a respondent's household is registered might be a factor affecting health. Hukou was a dummy variable. The urban household registration was coded “1,” whereas rural household registration was coded “0.”

**Marital status:** Establishing a marital relationship may alleviate health problems (53), and married women may obtain help in housework and childcare from their husbands. Therefore, in this study marital status was binary and divided into the following two categories: Married and other (including the status of separated, divorced, widowed, and never married). Marital status was coded “1” if the answer was “married” and “0” otherwise.

**Father and mother:** Women of childbearing age have older parents to help with their housework and child care, which is important to alleviate work-family contradiction and good for their health to a certain extent (54). Older parents are living and in good health, which is the premise of taking care of grandchildren (55). The control variables of father and mother were obtained from the questions: “Is your father/mother still living and in good health?” The answer of “Yes” was coded as “1”; otherwise “0.”

**Migrant experience:** The migrant women generally have limited access to full labor rights, limited medical consumption capacity, and exclusion from the formal health system of the area where they migrated (52, 56). Thus, the migrant experience would be a factor affecting health. The migrant experience was obtained from the question: “Have you leaved the area of residence registration for more than half a year?” The answer of “Yes” was coded as “1”; otherwise, 0. In the empirical part, we controlled the regional effect according to the provinces where the respondents were located. The descriptive analysis of these variables is provided in Table 1.

**Table 1:** Descriptive statistics.

**Variable**	**Mean**	**SD**	**Min**.	**Max**.
Physical health	3.747	0.907	1	5
BMI	0.415	0.493	0	1
Mental health	27.888	8.576	20	80
Total number of births	1.793	0.776	1	7
Logarithm of income	10.243	1.155	1.609	14.914
Multiple roles	0.863	0.344	0	1
Gender of the first child	0.449	0.497	0	1
Age	41.057	7.191	18	49
Education	9.212	4.000	0	19
Smoking	0.299	0.458	0	1
Drinking	0.214	0.410	0	1
Exercise	0.317	0.465	0	1
Father	0.425	0.494	0	1
Mother	0.521	0.500	0	1
Migrant experience	0.117	0.322	0	1
**Variable**	**Item**	**Freq**.	**Precent**	
Hukou	Urban	879	20.71	
	Rural	3,366	79.29	
Marital status	Married	4,007	94.39	
	Other	238	5.61	
*N*		4,245				

### Empirical Model

As the physical health of the sampled women is represented by the discrete and ordered data, the ordered probit model can be used to estimate the effect of fertility on the physical health of women of childbearing age. The ordered probit model can be written as follows:


(1) $$\begin{array}{r} {Physical\;health}^{*}=\;\alpha_{0}\;+\;\beta x+\;\gamma Z\;+\;\varepsilon , \end{array}$$

where physical health^*^ is the explained variable, representing the physical health of women of childbearing age; α_0_ is the intercept term; *x* is the explanatory variable, namely, total number of births; *Z* represents control variables; β and γ are coefficient vectors; and ε is an error term.

In the model, Physical health^*^ cannot be observed directly, whereas *Physical health* can be observed. The selection rule of *Physical health* is as follows:


(2) $$Physical\;health\;=\left\{ \begin{array}{l} \;=1\;If\;Physical\;health*\leq \;1\\ =2\;1<Physical\;health^{*}\leq \;\mu_{1}\\ =3\;\mu_{1}<Physical\;health^{*}\leq \;\mu_{2}\\ \;\cdots \;\cdots \;\\ \;=J\;Physical\;health^{*}\geq \;\mu_{J-2} \end{array}\right.$$

where μ_1_ < μ_2_ < ⋯ ≤ μ_*J*−2_ are unknown parameters estimated together with β and γ.

The mental health of women of childbearing age was a continuous variable, and the ordinary least squares (OLS) model was employed to estimate the effect of fertility on the sampled women's mental health. The OLS model can be written as follows:


(3) $$\begin{array}{r} Mental\;health={\;\alpha }_{1}\;+\;\delta x\;+\;\vartheta Z\;+\;\eta , \end{array}$$

where *Mental* health is the explained variable, meaning the mental health of women of childbearing age assessed by the CES-D scale; *x* and *Z* have the same meaning as in Equation (1); α_1_ is the intercept term; δ and ϑ are coefficients; and η is the error term.

Although the ordered probit and OLS models can indicate the effects of fertility on the sampled women's physical and mental health to a certain extent, the endogeneity is still a considerable problem that needs to be solved. Although most of the factors influencing health are controlled, the endogeneity caused by potential confounding factors cannot be ignored. To address the endogeneity problem, the IV-ordered probit and the IV two-stage least squares (IV-2SLS) models were employed (57).

We chose the gender of the first child as the IV. Theoretically, the gender of the first child is qualified to meet the requirements of relevance and exclusion of the IV. For relevance, because of the pursuit of the patriarchal family system and the relative liberalization of the birth policy, parents whose first child is a girl are more likely to have more children. As such, women's total number of births might be closely related to gender of the first child. For exclusion, the gender of the child is almost random, which does not directly affect women's health. Therefore, the IV satisfies the relevance and exclusion requirements. The IV in this study was a binary variable. The gender of the first child of respondent is girl was code as “1”; otherwise “0.”

The IV was subjected to diagnostic tests. According to weak IV identification tests, the Cragg–Donald Wald F statistic and Kleibergen–Paap rk Wald F statistic were 210.909 and 206.395, respectively, which were both significantly higher than 16.38, the critical value of the Stock–Yogo weak IV test (10%). Thus, the null hypothesis that the IV was weak was not supported. The Kleibergen–Paap rk LM statistic was 196.973 and significant at the 1% significance level, which rejected the null assumption of the under-identification and revealed that the IV closely related to the endogenous variable, the total number of births. Furthermore, in the first regression stage, the *F* value was 206.390. In sum, the IV theoretically and empirically meets the relevance and exclusion requirements, and the results estimated by the IV approach are reliable.

Except for the IV approach, we further employed the IPTW-RA method for managing multiple treatment groups and overcoming the selective bias. The propensity score technique is a widespread method to eliminate the pretreatment imbalances on observed variables and examine the effects of treatments or interventions. The propensity score was mostly used to control for imbalances and compared two treatment groups of interest (i.e., groups of treatment and control) (58). Nonetheless, the propensity score techniques can be extended to three multiple treatment groups (i.e., the groups of treatment A, treatment B, and control) or more (59).

The IPTW-RA is a joint model composed of inverse probability weighted model and regression adjustment model, and it can manage multiple treatment groups (59). It is a dual robust estimation method. If only the endogenous problem under observable variables is considered, the consistent estimation can be obtained once one of the outcome model or treatment model is set correctly (60). Therefore, it is a more effective estimation method among various methods to overcome selective bias, and the results of the IPTW-RA is more robust and suitable for the estimation of multiple treatment groups (60). Therefore, in this study, we categorized the sampled women into three groups (i.e., women with one child, women with two children, and women with three and more children), and assessed between-group differences in physical and mental health outcomes of the sampled women by using the IPTW-RA after balancing groups.

Moreover, 1,000 times bootstrap estimation was used to test the mediating effects of income and multiple roles in the influence of fertility on physical and mental health of women of childbearing age. The basic principle underlying the test for mediating effects in the bootstrap approach is that the standard error estimation and confidence interval (CI), which are calculated based on the assumption of a normal distribution, are imprecise as the indirect effect estimation generally do not follow normal distribution (61). MacKinnon et al. argued that the usage of bootstrap estimation can control the estimation errors and provide the CIs with good statistical capacity (62).

To test the mediation effect, the bootstrap estimation draws many bootstrap samples (generally, 1,000). The indirect effect is estimated for each bootstrap sample, and a bootstrap distribution of the estimated indirect effect is calculated. If the 95% CI of the bootstrap distribution does not overlap with zero, a null hypothesis can be rejected and then the mediating effect is confirmed (61). The STATA 15.0 (StataCorp. LP., College Station, TX, USA) was used for all statistical analyses.

## Results

### Descriptive Analysis

Table 1 shows the results of the descriptive analysis (*N* = 4,245). For the explained variables, the average value for physical health was 3.747 (SD = 0.907) on a five-point Likert scale of 1–5. The objective index of BMI had an average value of 0.415 (SD = 0.493), which indicated that 41.5% of the respondents' BMI were in normal range. The mean value for mental health was 27.888 (SD = 8.576) on a scale of 20–80. The explanatory variable, the total number of births, ranged from 1 to 7, with a mean value of 1.793 (SD = 0.776).

In terms of the mediating variables, the logarithm of income had a mean value of 10.243 (SD = 1.155) and ranged from 1.609 to 14.914. The variable of multiple roles ranged from 0 to 1 and had a mean value of 0.863 (SD = 0.344). The IV, gender of the first child had a mean value of 0.449 (SD = 0.497), which suggested that 44.9% of the sampled women's the first child was a daughter.

Among the control variables, age ranged from 18 to 49 years, with a mean value of 41.057 (SD = 7.191). The average amount of education was 9.212 (SD = 4.000) years, with a minimum value of 0 years (illiteracy) and a maximum value of 19 years (master's). In terms of lifestyles, 29.9% of the sampled women smoked, 21.4% drank alcohol, and 31.7% exercised regularly. The 42.5 and 52.1% of the respondents' fathers and mothers were still living and in good health, respectively. The 11.7% of the sampled had half of a year migrant experience. A total of 20.71% of sampled women's households (Hukou) were registered in an urban area, and 94.39% were married.

### Benchmark Regression

The ordered probit and OLS models were employed for benchmark regressions to estimate the effects of fertility on the physical and mental health of women of childbearing age, respectively, and the results are shown in Table 2. The results in columns 1 and 2 of Table 2 reported the effect of fertility on physical health of women of childbearing age. The results in column 1 were estimated without controls, whereas the results in column 2 were estimated after adding all controls. The results in both columns 1 and 2 showed that the total number of births was significantly and negatively associated with the sampled women's physical health (β = −0.151, *p* < 0.01; β = −0.082, *p* < 0.01).

**Table 2:** Effects of fertility on physical and mental health of women of childbearing age.

**Variable**	**Physical health**		**Mental health**	
	**(Ordered probit)**		**(OLS)**	
	**(1)**	**(2)**	**(3)**	**(4)**
Total number of births	−0.151***	−0.082***	2.830***	2.532***
	(0.021)	(0.023)	(0.164)	(0.176)
Age		−0.017***		−0.012
		(0.002)		(0.019)
Education		0.010**		−0.090**
		(0.005)		(0.039)
Smoking		0.068*		−0.822***
		(0.040)		(0.301)
Drinking		0.054		0.691**
		(0.044)		(0.335)
Exercise		0.100***		−0.433
		(0.038)		(0.284)
Hukou		0.009		−0.186
		(0.041)		(0.314)
Marital status		0.069		−0.718
		(0.072)		(0.550)
Father		0.035		−0.647**
		(0.040)		(0.306)
Mother		−0.001		0.170
		(0.040)		(0.303)
Migrant experience		−0.170***		0.084
		(0.053)		(0.400)
Logarithm of income		0.105***		−0.626***
		(0.016)		(0.125)
Province	Yes	Yes	Yes	Yes
Pseudo *R*^2^	0.012	0.028	–	–
Adj-*R*^2^	–	–	0.085	0.097
*N*			4,245	

Standard errors in the parentheses; ***p < 0.01, **p < 0.05, *p < 0.1.

The results in column 3 without controls and column 4 with all controls added were similar, and they both demonstrated that the total number of births was significantly correlated with the mental health of women of childbearing age (δ = 2.830, *p* < 0.01; δ = 2.532, *p* < 0.01). This finding indicated that the higher the number of births, the greater the score on the CES-D scale, and the worse the mental health status of women of childbearing age.

The results in Table 2 confirmed the negative effects of fertility on the physical and mental health of women of childbearing age and preliminarily demonstrated the existence of the motherhood health penalty in China. However, the problem of endogeneity was not solved. Thus, we employed the IV to address it in next section.

### Instrumental Variable Approach

The results in Table 3 presented the impacts of fertility on the physical and mental health of women of childbearing age after addressing the problems of endogeneity. As observed in columns 1 and 2 in Table 3, by employing IV-ordered probit models, the total number of births was significantly and negatively related to the sampled women's physical health (β = −0.271, *p* < 0.01; β = −0.204, *p* < 0.01). This meant that after solving the endogeneity problem, the impact of fertility on the physical health of women of childbearing age was still negative.

**Table 3:** Impacts of fertility on physical and mental health of women of childbearing age.

**Variable**	**Physical health**		**Mental health**	
	**(IV-Ordered probit)**		**(IV-2SLS)**	
	**(1)**	**(2)**	**(3)**	**(4)**
Total number of births	−0.271***	−0.204***	2.990***	2.833***
	(0.067)	(0.072)	(0.806)	(0.804)
Age		−0.017***		−0.012
		(0.002)		(0.019)
Education		0.006		−0.073
		(0.006)		(0.059)
Smoking		0.066*		−0.858***
		(0.040)		(0.316)
Drinking		0.062		0.708**
		(0.043)		(0.337)
Exercise		0.078**		−0.400
		(0.037)		(0.297)
Hukou		0.009		−0.192
		(0.040)		(0.314)
Marital status		0.108		−0.816
		(0.074)		(0.606)
Father		0.032		−0.639**
		(0.039)		(0.307)
Mother		−0.001		0.160
		(0.039)		(0.304)
Migrant experience		−0.178***		0.077
		(0.052)		(0.400)
Logarithm of income		0.094***		−0.610***
		(0.017)		(0.132)
atanhrho_12	0.094*	0.089*	–	–
	(0.054)	(0.053)	–	–
Province	Yes	Yes	Yes	Yes
*R* ^2^	–	–	0.085	0.099
Weak IV identification test	Cragg–Donald Wald F statistic		210.909	
	Kleibergen–Paap rk Wald F statistic		206.395	
Under–identification test	Kleibergen–Paap rk LM statistic		196.973	
*F*-value of the first stage	206.390			
*N*	4,245			

Standard errors in the parentheses; ***p < 0.01, **p < 0.05, *p < 0.1.

Similarly, the results in columns 3 and 4, which were estimated by employing IV-2SLS models, indicated that the total number of births was significantly associated with the sampled women's mental health (δ = 2.990, *p* < 0.01; δ = 2.833, *p* < 0.01). The results suggested that after endogeneity was addressed, the conclusion that a higher number of births was associated with a higher CES-D score and thus poorer mental health among women of childbearing age remained valid.

### Inverse Probability of Treatment Weighting and Regression Adjustment

Table 4 provides the average treatment effect (ATE) estimated by the IPTW-RA method after dealing with the self-selectivity. The results in Table 4 showed that for the physical health, the ATEs of the women with two children and women with three and more children were −0.075 (*p* < 0.05, CI: from −0.137 to −0.013) and −0.139 (*p* < 0.01, CI: from −0.237 to −0.042), respectively. It indicated that compared to the women with one child, the women with two children and the women with three and more children had the worse physical health. Thus, the effect of fertility on the physical health of women of childbearing age was still significant and negative.

**Table 4:** Results of inverse probability of treatment weighting regression adjustment (IPTW–RA).

	**ATE**	**95% CI**	
		**Lower**	**Upper**
**Physical health**			
Two children vs. One child	−0.075**	−0.137	−0.013
	(0.032)		
Three and more children vs. One child	−0.139***	−0.237	−0.042
	(0.050)		
**Mental health**			
Two children vs. One child	−0.275	−0.872	0.321
	(0.346)		
Three and more children vs. One child	5.246***	4.490	6.000
	(0.384)		

Robust standard errors in the parentheses; ***p < 0.01, **p < 0.05. ATE means the average treatment effect, and CI is the confidence interval. The treatment model is Multinomial Logit Model (MLN). After weighted, the covariates passed the balance test.

For mental health, as shown in Table 4, the ATE of women with two children had an insignificant value of −0.275 (CI: from −0.872 to 0.321), and that of women with three children had a significant value of 5.246 (*p* < 0.01, CI: from 4.490 to 6.000). These results suggested that women with two children did not show a significant worse mental health compared with women with one child, but women with three children observed a significant worse mental health. The results estimated by the IPTW-RA method were roughly consistent with the results using the baseline models and IV approach.

### Heterogeneity Analysis

As mentioned in the Control Variables section, there is a large disparity between urban and rural areas in China, and the medical care that women receive is closely related to the area where their household is registered (51, 52). Therefore, there may be significant differences in the impact of fertility on the health of women whose households are registered in different regions. We grouped the entire sample into two subgroups according to the area where the sampled women's households were registered: The urban samples and rural samples.

The results in Table 5 showed the different impacts of fertility on women's physical and mental health with the use of IV-ordered probit and IV-2SLS models, respectively. It can be observed in Table 5 that the total number of births was significantly and negatively associated with rural women's physical health (β = −0.210, *p* < 0.01), whereas the results of urban samples were insignificant. Furthermore, the total number of births was also significantly related to rural women's mental health (δ = 2.758, *p* < 0.01), but the results of urban samples were insignificant. The results indicated that compared with urban women, who may receive better medical treatment, rural women's physical and mental health may be more substantially and easily impacted by fertility.

**Table 5:** Heterogenous impacts of fertility on physical and mental health of women of childbearing age.

**Variable**	**Physical health**		**Mental health**	
	**(IV-Ordered probit)**		**(IV-2SLS)**	
	**Urban**	**Rural**	**Urban**	**Rural**
Total number of births	−0.170	−0.210***	3.066	2.758***
	(0.165)	(0.080)	(2.015)	(0.869)
Age	−0.018***	−0.016***	0.026	−0.024
	(0.005)	(0.003)	(0.042)	(0.021)
Education	−0.015	0.013*	0.052	−0.113*
	(0.014)	(0.007)	(0.148)	(0.063)
Smoking	0.015	0.078*	−0.799	−0.895**
	(0.088)	(0.045)	(0.728)	(0.349)
Drinking	0.131	0.048	−0.081	0.900**
	(0.094)	(0.049)	(0.765)	(0.377)
Exercise	0.180**	0.053	−0.084	−0.509
	(0.081)	(0.042)	(0.644)	(0.333)
Marital status	0.200	0.080	−3.593**	−0.085
	(0.166)	(0.083)	(1.465)	(0.664)
Father	0.060	0.019	0.024	−0.786**
	(0.085)	(0.044)	(0.677)	(0.343)
Mother	−0.006	0.001	−0.096	0.226
	(0.087)	(0.044)	(0.688)	(0.338)
Migrant experience	−0.240*	−0.190***	−1.236	0.522
	(0.123)	(0.057)	(1.022)	(0.440)
Logarithm of income	0.037	0.105***	0.049	−0.729***
	(0.038)	(0.018)	(0.310)	(0.146)
atanhrho_12	0.067	0.094	–	–
	(0.118)	(0.059)	–	–
Province	Yes	Yes	Yes	Yes
R^2^	–	–	0.073	0.114
*N*	879	3,366	879	3,366

Standard errors in the parentheses; ***p < 0.01, ^**^p < 0.05, *p < 0.1.

### Robustness Check

#### Replacing Data and Teasing out Effect of Abortion

Women's abortion experience is a factor should be considered when looking at the impact of fertility on women's physical and mental health. However, the CLDS2018 did not involve the information related women's abortion experience. We further employed the CLDS2016 dataset which concerned women's abortion experience to control the effect of abortion experience on women's health and test the robustness of the results. The variable of abortion experience was obtained in the CLDS2016 by the question of “Have you ever had an abortion experience?” The answer of “Yes” was coded as “1”; otherwise “0.” The results of replacing data and teasing out effect of abortion were provided in Table 6.

**Table 6:** Robustness check of using the CLDS2016 and teasing out the effect of abortion.

**Variable**	**Physical health**		**Mental health**	
	**Ordered probit**	**IV–ordered probit**	**OLS**	**IV−2SLS**
Total number of births	−0.535***	−0.742***	1.250***	1.904***
	(0.055)	(0.093)	(0.412)	(0.709)
Age	−0.024***	−0.022***	−0.055	−0.061
	(0.007)	(0.007)	(0.052)	(0.053)
Education	−0.012	−0.019	−0.113	−0.090
	(0.012)	(0.012)	(0.093)	(0.092)
Smoking	−0.800***	−0.818***	5.714***	5.795**
	(0.272)	(0.271)	(2.127)	(2.712)
Drinking	0.267	0.285	−2.241	−2.305**
	(0.209)	(0.209)	(1.612)	(1.066)
Exercise	0.059	0.031	−1.115	−1.027
	(0.093)	(0.094)	(0.719)	(0.705)
Hukou	−0.081	−0.101*	0.020	0.083
	(0.056)	(0.056)	(0.434)	(0.389)
Marital status	0.429**	0.443**	−3.600**	−3.663
	(0.212)	(0.211)	(1.646)	(2.276)
Father	0.025	0.029	−0.504	−0.520
	(0.085)	(0.085)	(0.659)	(0.645)
Mother	0.004	−0.010	−0.215	−0.174
	(0.096)	(0.096)	(0.748)	(0.748)
Migrant experience	0.070	0.140	−0.106	−0.322
	(0.110)	(0.113)	(0.856)	(0.865)
Abortion experience	−0.258***	−0.249***	2.217***	2.197***
	(0.092)	(0.092)	(0.712)	(0.756)
Logarithm of income	0.072*	0.052	−0.259	−0.199
	(0.040)	(0.040)	(0.306)	(0.319)
atanhrho_12	–	0.180***	–	–
	–	(0.069)	–	–
Province	Yes	Yes	Yes	Yes
Pseudo R^2^	0.075	–	–	–
R^2^	–	–	0.079	0.076
Weak IV identification test	Cragg–Donald Wald F statistic		337.550	
	Kleibergen–Paap rk Wald F statistic		575.856	
Under–identification test	Kleibergen–Paap rk LM statistic		230.980	
F value of the first stage	575.860		
*N*	823		

Standard errors in the parentheses; ***p < 0.01, **p < 0.05, *p < 0.1.

It can be observed from Table 6, when the dataset was replaced and the effects of abortion on women's health were controlled, the baseline regression results of ordered probit and OLS models showed that total number of births was significantly associated with physical (β = −0.535, *p* < 0.01) and mental health (δ = 1.250, *p* < 0.01). The results of IV-ordered probit and IV-2SLS models reported that the total number of births significantly related to physical health (β = −0.742, *p* < 0.01) and mental health (δ =1.904, *p* < 0.01) of women of childbearing age. Thus, by replacing the dataset and teasing out the effect of abortion, the results in this study were stable.

#### Relaxing Instrument Exogeneity

As the second-best way to test the robustness of the results, assuming that the IV—the variable of gender of the first child failed to satisfy the usual exogeneity condition, we tested the parameter sensitivity following Conley et al. (63) plausibly exogenous estimation method. Table 7 reported the plausibly exogenous estimation results of union of confidence intervals (UCI) and local to zero (LTZ) methods.

**Table 7:** Conley et al. (63) plausibly exogenous bounds.

**Variable**	**UCI**		**LTZ**	
	**Physical**	**Mental**	**Physical**	**Mental health**
	**health**	**health**	**health**	**health**
Total number of births	(−0.367 to −0.027)	(1.255 to 4.397)	(−0.363 to −0.024)	(1.271 to 4.401)
Control variables	Yes	Yes	Yes	Yes
Province	Yes	Yes	Yes	Yes

*The estimator of the lower bound and the upper bound for the coefficient of total number of births is shown in parentheses with a 95% significance level*.

For the physical health, the bounds on the endogenous variable of women's total number of births estimated with the UCI method were (−0.367 to −0.027), and the bounds estimated with the LTZ method were (−0.363 to −0.024). For mental health, the bounds estimated by UCI and LTZ methods were (1.255−4.397) and (1.271-4.401), respectively. The coefficients of β, −0.204 (physical health) and δ, 2.833 (mental health) estimated in the IV regression were both within the corresponding intervals in the UCI and LTZ methods. It was meant that although the exogeneity of the instrument was relaxed, our results of the IV approach were still valid.

#### Alternative Explained Variable

To overcome the bias associated with self-rated health classifications, the variable of BMI was applied to estimate the impact of fertility on physical and mental health of women of childbearing age. As the variable of BMI was a binary variable, the probit model was suitable for estimation. The probit and IV-probit regression results are displayed in Table 8. It can be observed from Table 8 that the coefficients of total number of births were negative and significant in the probit model (coefficient = −0.080, *p* < 0.01) and IV-probit model (coefficient = −0.322, *p* < 0.01).

**Table 8:** Robustness check of alternative explained variable BMI.

**Variable**	**BMI**	
	**Probit**	**IV–probit**
Total number of births	−0.080***	−0.322***
	(0.028)	(0.120)
Age	−0.009***	−0.009***
	(0.003)	(0.003)
Education	0.006	−0.007
	(0.006)	(0.009)
Smoking	0.118**	0.146***
	(0.047)	(0.048)
Drinking	0.005	−0.009
	(0.052)	(0.052)
Exercise	0.082*	0.054
	(0.044)	(0.046)
Hukou	0.006	0.012
	(0.049)	(0.049)
Marital status	0.064	0.141
	(0.086)	(0.094)
Father	0.117**	0.110**
	(0.048)	(0.048)
Mother	−0.005	0.003
	(0.048)	(0.047)
Migrant experience	−0.092	−0.086
	(0.062)	(0.062)
Logarithm of income	0.075***	0.061***
	(0.020)	(0.021)
Province	Yes	Yes
Pseudo *R*^2^	0.022	–
Wald test	–	4.040** (*p* <0.05)
*N*	4,245	

Standard errors in the parentheses; ^***^p < 0.01, **p < 0.05, *p < 0.1.

Overall, the results of the series of robustness checks indicated that total number of births did have a negative impact on physical and mental health of women of childbearing age, and the existence of the motherhood health penalty in China can be demonstrated.

### Mechanism Analysis

After the negative impacts of fertility on the physical and mental health of women of childbearing age were verified, the potential mechanisms through which fertility affected the physical and mental health of women of childbearing were tested. The bootstrap estimation procedure was applied to test the significance of the mediating effects of income and multiple roles. Bootstrap samples were drawn 1,000 times to test the mediating effects. The 95% CIs of the indirect effects are shown in Table 9. It can be concluded from Table 9 that the model pathways of Total number of births → Income → Physical health, Total number of births → Income → Mental health, Total number of births → Multiple roles → Physical health, and Total number of births → Multiple roles → Mental health were all significant.

**Table 9:** Mediating effects of income and multiple roles in fertility effects on physical and mental health of women of childbearing age.

**Model pathway**	**Indirect effect**	**Percentile 95% CI**	
		**Lower**	**Upper**
Total number of	−0.010***	−0.013	−0.002
births → Income → Physical health	(0.003)		
Total number of	0.101***	0.054	0.157
births → Income → Mental health	(0.026)		
Total number of births → Multiple	−0.008***	−0.013	−0.002
roles → Physical health	(0.003)		
Total number of births → Multiple	0.092***	0.041	0.144
roles → Mental health	(0.027)		

Bootstrap standard errors in the parentheses; ***p < 0.01. CI means confidence interval. The control variables were included in the process of bootstrapping.

Furthermore, no corresponding 95% CIs overlapped with zero. It can be concluded that the variables of income and multiple roles mediated the impacts of the total number of births on the physical and mental health of Chinese women of childbearing age. The results proved that the fertility impacted the physical and mental health of women of childbearing age through the potential channels of limited family resources and the multiple roles played by mothers.

## Discussion

In 2015, the United Nations adopted 17 sustainable development goals (SDGs) to be achieved by 2030, including gender equality (64). The motherhood penalty is a crucial topic in the field of gender equality. In this study, focusing on Chinese women of childbearing age, we employed nationally representative survey data (CLDS2018), examined the effects of fertility on mothers' physical and mental health, and demonstrated the motherhood health penalty in China. The findings are as discussed in the following.

First, after addressing endogeneity, the results estimated by using the IV-ordered probit model and IV-2SLS model indicated that, in general, the greater the total number of births, the worse the physical and mental health of women of childbearing age. The results of IPTW-RA method showed that although women with two children did not have a significant worse mental health compared with women with one child, the women with three children had a significant worse mental health. Having more children means that the women experience more childbearing life events. This exerts stress on physical functions and leads to the accumulation of health disadvantages with adverse effects on women's health. These health sacrifices made by women are irreversible to a large extent and have lasting negative effects (65). The financial support and accompanying care provided by multiple children to women in their old age may not compensate for the health sacrifices they made during their childbearing years.

Second, the results of heterogeneity analysis showed that fertility more easily affected the physical and mental health of rural women compared with urban women. In China, there are evident development disparities being addressed between rural and urban areas, including gaps in income, social security, education, and infrastructure (66–68). Compared with urban women, rural women may experience poorer reproductive healthcare and medical treatment. In addition, to provide better financial support for families, rural males in the labor force transfer to urban areas for work, resulting in the phenomenon of rural left-behind women. The number of left-behind wives in Chinese rural areas reached 47 million in 2019 (69). As such, rural women may shoulder the accumulated pressures of housework, farming, and taking care of children alone, which has serious negative impacts on their physical and mental health.

Third, in the mechanism analysis, income and multiple roles were found to mediate the impacts of fertility on the physical and mental health of women of childbearing age. Women's income is a vital financial resource to support their healthcare, and the motherhood income penalty has been demonstrated by extensive research in different cultural contexts. In the United States, for every child born, the mothers experience a 7% reduction in income (70); in the United Kingdom and Germany, every child that a woman bears decreases her income by about 9% (71). In China, women's income decreases by about 17% in the year of childbirth (72). The low income of Chinese women of childbearing age has led to a decline in their living standards and poor health. Furthermore, as the traditional primary caregiver of the family, women often shoulder labor-intensive family responsibilities, such as performing household chores and educating children. Most women today have their own careers, so they also experience the professional pressure of the workplace. The adverse influences on the physical and mental health of women arising from their multiple roles cannot be neglected.

From these findings, some policy implications can be drawn to mitigate the motherhood health penalty in China and other developing counties or regions. First, in addition to paying attention to the demographic dividend and economic development resulting from increased rates of childbirth, the government should also focus on the health of women of childbearing age. In particular, the three-child policy announced in May 2021 provides opportunities for women to have more children, but it may also increase uncertainty and risks to their health and exacerbate the effects of the health penalty. As we proved, the third child can bring about women's significant worse physical and mental health. Therefore, under the condition of birth policy relaxing, maternity leave for mothers and paternity leave for fathers should be stipulated by the government and implemented efficiently across industries and departments. Moreover, there were about 50 million infants under 3 years old in China, but the nursery school enrollment rate of children under 3 years old was <5% in 2020 (73). The institutional arrangements of childcare services, which can reduce the physical and mental pressure on mothers, can be substantially strengthened.

Second, to improve the physical and mental health of rural women of childbearing age, the government can vigorously develop medical facilities and enhance the professional ability of medical personnel in rural areas. The government can accelerate rural vitalization and encourage workers in the labor force to remain in the countryside, build their hometown, and share the pressures of housework, farming, and raising children with their wives. In addition, the government can also regularly carry out physical health examinations and mental health counseling for women in rural areas.

Third, to ensure the income of women of childbearing age, the working and family-friendly policy can be improved and refined. Compared with developed countries, the working and family-friendly policy for women in China is still in its infancy (74). An employer can create a favorable and flexible workplace atmosphere for mothers, for instance, by allowing them to work from home to ensure that they earn an income and help them to cope with conflicts between family and work. The government can institutionalize anti-discrimination measures to protect mothers' employment and emphasize the empowerment of trade unions to strengthen mothers' bargaining power in the labor market. Furthermore, to relieve mothers' stress caused by their multiple roles, fathers should be encouraged to participate in family chores and childcare. There is a social phenomenon known as “widowed child-rearing” in China (75). This term refers to the mother providing emotional and educational support to the children while the father plays a minimal role in childcare (75). Therefore, to overcome the health problems caused by the pressure of multiple roles, it is necessary to encourage fathers to participate in family affairs and childcare.

There are some limitations in this study and possible directions for further studies. First, although we used the IV approach to address the endogeneity issue produced by potential confounding variables, it is undeniable that being a mother also leads to happiness and self-fulfillment in women. Thus, motherhood may be conducive to women's health outcomes. This positive effect of motherhood on women of childbearing age was not considered in this study due to the limitation of the dataset. Therefore, if data are available, further research can try to address this limitation.

Second, in addition to the extensive discussions of the motherhood wage penalty, the fatherhood wage premium has also received much attention. By comparing the health status of fathers and mothers, the gender-based health inequality caused by childbearing may be revealed. Therefore, future research focusing on the motherhood health penalty can examine the existence of the fatherhood health premium.

Third, as the CLDS did not involve the questions related to respondents' husband such as husbands' time consumed in doing housework and childcare, we controlled the marital status of women of childbearing age to control the influence of husbands as much as possible. The further study can empirically test the effect of husbands' time consuming in housework and childcare on physical and mental health of women of childbearing if the data is available.

## Conclusions

This study empirically clarifies the relationship between fertility and the health outcomes of women of childbearing age in China. The results of IV approach indicated that fertility had significant adverse impacts on mothers' physical and mental health, which demonstrated the existence of the motherhood health penalty in China. The results of IPTW-RA method suggested that although women with two children did not have a significant worse mental health compared with women with one child, the women with three children had a significant worse mental health. The results of heterogeneity analysis showed that the impacts of fertility on physical and mental health differ between rural and urban mothers. The income and multiple roles were demonstrated to play mediating roles in the impacts of fertility on the physical and mental health of women of childbearing age. The findings of this study extended the motherhood penalty from the labor market to the health domain and had important implications for improving the healthcare policy for women of childbearing age in China and other countries and regions.

## Data Availability Statement

The data analyzed in this study are available from the corresponding author on reasonable request.

## Ethics Statement

An informed consent was obtained from all subjects involved in the study.

## Author Contributions

YJ proposed the idea of this study, developed the method, and wrote the theoretical analysis, results, discussion, and conclusions. FY gave guidance in the theory, and edited the whole manuscript. All authors have read and agreed to the published version of the manuscript.

## Conflict of Interest

The authors declare that the research was conducted in the absence of any commercial or financial relationships that could be construed as a potential conflict of interest.

## Publisher's Note

All claims expressed in this article are solely those of the authors and do not necessarily represent those of their affiliated organizations, or those of the publisher, the editors and the reviewers. Any product that may be evaluated in this article, or claim that may be made by its manufacturer, is not guaranteed or endorsed by the publisher.

## References

[1] Kmec JA. Are motherhood penalties and fatherhood bonuses warranted? Comparing pro-work behaviors and conditions of mothers, fathers, and non-parents. Soc Sci Res. (2011) 40:444–59. 10.1016/j.ssresearch.2010.11.006

[2] Kelley H Galbraith Q. Strong J. Working moms: motherhood penalty or motherhood return? J Acad Librarian. (2020) 46:102075. 10.1016/j.acalib.2019.102075

[3] Baker M. Motherhood, employment and the “child penalty”. Womens Stud Int Forum. (2010) 33:215–24. 10.1016/j.wsif.2010.01.004

[4] Cukrowska-Torzewska E Matysiak A. The motherhood wage penalty: a meta-analysis. Soc Sci Res. (2020) 88–89:102416. 10.1016/j.ssresearch.2020.102416 32469733

[5] Nizalova OY Sliusarenko T Shpak S. The motherhood wage penalty in times of transition. J Compar Econ. (2016) 44:56–75. 10.1016/j.jce.2015.10.009

[6] Berniell I Berniell L María Edo DM Marchionni M. Gender gaps in labor informality: the motherhood effect. J Dev Econ. (2021) 150:102599. 10.1016/j.jdeveco.2020.102599

[7] Conde-Agudelo A Rosas-Bermúdez A Kafury-Goeta AC. Effects of birth spacing on maternal health: a systematic review. Am J Obstetr Gynecol. (2007) 196:297–308. 10.1016/j.ajog.2006.05.055 17403398

[8] Winikoff B. The effects of birth spacing on child and maternal health. Stud Fam Plan. (1983) 14:231–45. 10.2307/1965748 6648993

[9] Ganatra B Faundes A. Role of birth spacing, family planning services, safe abortion services and post-abortion care in reducing maternal mortality. Best Pract Res Clin Obstetr Gynaecol. (2016) 36:145–55. 10.1016/j.bpobgyn.2016.07.008 27640082

[10] Zhou C Weng J Tan F Wu S Ma J Zhang B. Pregnancy-related anxiety among Chinese pregnant women in mid-late pregnancy under the two-child policy and its significant correlates. J Affect Disord. (2020) 276:272–78. 10.1016/j.jad.2020.07.099 32697709

[11] Liu Y Hu J. The more children raised, the more blessings of mother: the impact of number of children on maternal health. South China Popul. (2016) 6:69–78. (in Chinese). 10.3969/j.issn.1004-1613.2016.06.007

[12] Peters SA Yang L Guo Y Chen Y Bian Z Millwood IY. Parenthood and the risk of cardiovascular diseases among 0.5 million men and women: Findings from the China Kadoorie Biobank. Int J Epidemiol. (2017) 1:180–9. 10.1093/ije/dyw144 27649806 PMC5837253

[13] Hankinson SE Colditz GA Hunter DJ Manson JE Willett WC Stampfer MJ. Reproductive factors and family history of breast cancer in relation to plasma estrogen and prolactin levels in postmenopausal women in the nurses' health study (United States). Cancer Causes Control. (1995) 3:217–24. 10.1007/BF00051793 7612801

[14] Mishra GD Cooper R Kuh D. A life course approach to reproductive health: theory and methods. Maturitas. (2010) 65:92–7. 10.1016/j.maturitas.2009.12.009 20079587 PMC3504662

[15] Kington R Lillard L Rogowski J. Reproductive history socioeconomic status and self-reported health status of women aged 50 years or older. Am J Public Health. (1997) 1:33–7. 10.2105/AJPH.87.1.33 9065223 PMC1380761

[16] Zhou Y Jia N Yang T. The quantity–quality trade-off related to investment in healthy human capital: new evidence from the implementation of the “selective two-child policy” in China. J Asian Econ. (2021) 76:101347. 10.1016/j.asieco.2021.101347

[17] Wu X Li L. Family size and maternal health: evidence from the One-Child policy in China. J Popul Econ. (2012) 25:1341–64. 10.1007/s00148-011-0361-0

[18] Liu Y Hu J. The number of children, parents' health and life satisfaction: the perspective of sex imbalance. Northwest Popul J. (2017) 3:68–75. (in Chinese). 10.15884/j.cnki.issn.1007-0672.2017.03.010

[19] Xu J Huang Y. Gender identity, marriage and labor behavior within households. Econ Res J. (2018) 4:135–50. (in Chinese)

[20] Fan X Im J Miao L Tomas S Liu H. Silk and steel: a gendered approach to career and life by upper echelon women executives in the hospitality and tourism industry in China. Int J Hospital Manag. (2021) 97:103011. 10.1016/j.ijhm.2021.103011

[21] Pugliese F. Mining companies and gender(ed) policies: the women of the Congolese Copperbelt, past and present. Extract Industr Soc. (2020) 8:100795. 10.1016/j.exis.2020.08.006

[22] Bernstein AB. Motherhood, health status, and health care. Womens Health Iss. (2001) 11:173–84. 10.1016/S1049-3867(01)00078-0 11336859

[23] Barnett RC Marshall NL. Worker and mother roles, spillover effects, and psychological distress. Women Health. (1992) 18:9–40. 10.1300/J013v18n02_02 1632103

[24] Sato S Liu Y Ikeda A Filomeno R Suzuki Y Maruyama K. Work-family conflict and insomnia symptoms among women working in aged care services in Japan. Sleep Med. (2021) 82:155–8. 10.1016/j.sleep.2021.03.034 33930791

[25] Štulhofer A Kuljanić K Buzina DS. Sexual health difficulties in a population-based sample of Croatian women aged 18–35 and the effects of the dual (career and motherhood) role. J Sex Med. (2011) 8:1314–21. 10.1111/j.1743-6109.2010.02100.x 21054803

[26] Tong X. Sociology of Population. Beijing: Peking University Press (2010). (in Chinese)

[27] National People's Congress (NPC). Population and Family Planning Law of the People's Republic of China. Available online at: http://www.npc.gov.cn/wxzl/wxzl/2001-12/30/content_282850.htm (accessed July 18, 2021).

[28] Wang Q Rizzo JA Fang H. Parents' son preference, childhood adverse experience and mental health in old age: evidence from China. Child Abuse Negl. (2019) 93:249–62. 10.1016/j.chiabu.2019.05.012 31129427

[29] Das Gupta M Jiang Z Li B Xie Z Chung W Bae HOK. Why is son preference so persistent in east and south Asia? A cross-country study of China, India, and the Republic of Korea. J Dev Stud. (2003) 40:153–87. 10.1080/00220380412331293807

[30] Mu Z Xie Y. “Motherhood penalty” and “fatherhood premium”? Fertility effects on parents in China. Demogr Res. (2016) 35:1373–410. 10.4054/DemRes.2016.35.47 30568537 PMC6296818

[31] Tian XY. The Policy of A Big Country: Review and Prospect of Population Policy of the People's Republic of China. Fuzhou: Fujian People's Publishing House (2020). (in Chinese)

[32] Peng P. China Family Planning Encyclopedia. Beijing: China Population Press (1997). (in Chinese)

[33] Wang F Cai Y. How are 400 million Chinese born less? China Reform. (2010) 7:85–88. (in Chinese).

[34] Zhang P Ma X Shi L. Migration, population aging, and income inequality in China. J Asian Econ. (2021) 76:101351. 10.1016/j.asieco.2021.101351

[35] Luo D Yan X Xu R Zhang J Shi X Ma J. Chinese trends in adolescent marriage and fertility between 1990 and 2015: a systematic synthesis of national and subnational population data. Lancet Global Health. (2020) 8:e954–64. 10.1016/S2214-109X(20)30130-3 32562651

[36] The State council the People's Republic of China. Decision on Implementing Comprehensive Two Child Policy Reform and Improving Family Planning Service Management. Available online at: http://www.gov.cn/gongbao/content/2016/content_5033853.htm (accessed July 18, 2021).

[37] The The State Council The The People's Republic of China. Available online at: http://www.gov.cn/xinwen/2021-07/20/content_5626190.htm (accessed July 18, 2021).

[38] Hare D. What accounts for the decline in labor force participation among married women in urban China, 1991–2011? China Econ Rev. (2016) 38:251–66. 10.1016/j.chieco.2016.01.004

[39] Zhang C Wang J. Gender roles and women's labor market outcomes. China Econ Quarter Int. (2021) 1:97–108. 10.1016/j.ceqi.2021.04.002

[40] *Marriage Law of the People's Republic of China*. Available online at: http://www.npc.gov.cn/wxzl/wxzl/2001-05/30/content_136774.htm (accessed July 18, 2021).

[41] Burki T. Health and rights challenges for China's LGBT community. Lancet. (2017) 389:1286. 10.1016/S0140-6736(17)30837-1 28379143

[42] United Nations. Age-Specific Fertility Rate, Total Fertility Mean Age at Childbearing. (2012). Available online at: https://www.un.org/en/development/desa/population/publications/dataset/fertiity/wfr2012/Metadata/Metadata_ASFR-TF-MAC.pdf (accessed July 18, 2021).

[43] WHO Expert Consultation. Appropriate body-mass index for Asian populations and its implications for policy and intervention strategies. Lancet. (2004) 363:157–63. 10.1016/S0140-6736(03)15268-3 14726171

[44] Rankin SH Galbraith ME Johnson S. Reliability and validity data for a Chinese translation of the Center for Epidemiological Studies–Depression. Psychol Rep. (1993) 73:1291. 10.2466/pr0.1993.73.3f.1291 8115582

[45] Zhang J Sun W Kong Y Wang C. Reliability and validity of the Center for Epidemiological Studies Depression Scale in 2 special adult samples from rural China. Comprehens Psychiatry. (2012) 53:1243–51. 10.1016/j.comppsych.2012.03.015 22520090 PMC3404200

[46] Yang F Jiang Y Paudel KP. Impact of credit constraints from formal financial institutions on rural residents' health in China. Healthcare. (2021) 9:6. 10.3390/healthcare9010006 33374650 PMC7822418

[47] Kahn RS Wise PH Kennedy BP Kawachi I. State income inequality, household income, and maternal mental and physical health: cross sectional national survey. Br Med J. (2000) 321:1311–5. 10.1136/bmj.321.7272.1311 11090512 PMC27533

[48] Yang F Jiang Y. Heterogeneous influences of social support on physical and mental health: evidence from China. Int J Env Res Public Health. (2020) 17:6838. 10.3390/ijerph17186838 32962140 PMC7558190

[49] Cui X Rockett IR Yang T Cao R. Work stress, life stress, and smoking among rural–urban migrant workers in China. BMC Public Health. (2012) 12:979. 10.1186/1471-2458-12-979 23151299 PMC3584974

[50] Gove WR. Sex, marital status, and mortality. Am J Sociol. (1973) 79:45–67. 10.1086/225505 4740470

[51] Song Q Smith JP. Hukou system, mechanisms, and health stratification across the life course in rural and urban China. Health Place. (2019) 58:102150. 10.1016/j.healthplace.2019.102150 31212169 PMC6708454

[52] Liu Z. Institution and inequality: the hukou system in China. J Compar Econ. (2005) 33:133–57. 10.1016/j.jce.2004.11.001

[53] Jiang Y Luo H Yang F. Influences of migrant construction workers' environmental risk perception on their physical and mental health: evidence from China. Int J Environ Res Public Health. (2020) 17:7424. 10.3390/ijerph17207424 33053832 PMC7601608

[54] Xiao X Loke AY. Intergenerational co-parenting in the postpartum period: a concept analysis. Midwifery. (2022) 107:103275. 10.1016/j.midw.2022.103275 35183874

[55] Zhen C Silverstein M. Caring for grandchildren and intergenerational support in rural China: a gendered extended family perspective. Ageing Soc. (2012) 32:425–50. 10.1017/S0144686X11000420

[56] Huang D. Medical treatment experiences of single rural-urban migrant women who induced abortion in mainland China: a qualitative study adopting social exclusion perspective. Asia Pac J Soc Work Dev. (2020) 30:260–72. 10.1080/02185385.2020.1735500

[57] Daniel D Pande S Rietveld L. Endogeneity in water use behaviour across case studies of household water treatment adoption in developing countries. World Dev Pers. (2020) 25:100385. 10.1016/j.wdp.2021.100385

[58] Stuart EA. Matching methods for causal inference: a review and a look forward. Stat Sci. (2010) 25:1–21. 10.1214/09-STS313 20871802 PMC2943670

[59] Imbens GW. The role of the propensity score in estimating dose-response functions. Biometrika. (2000) 87:706–10. 10.1093/biomet/87.3.706

[60] Cattaneo MD. Efficient Semiparametric estimation of multi-valued treatment effects under ignorability. J Econometr. (2010) 155:138–54. 10.1016/j.jeconom.2009.09.023

[61] Kong TZ He Auerbach RP McWhinnie CM Xiao J. Rumination and depression in Chinese university students: the mediating role of overgeneral autobiographical memory. Pers Individ Diff. (2015) 77:221–4. 10.1016/j.paid.2014.09.035 25977594 PMC4428603

[62] MacKinnon DP Lockwood CM Williams J. Confidence limits for the indirect effect: distribution of the product and resampling methods. Multivar Behav Res. (2004) 39:99–128. 10.1207/s15327906mbr3901_4 20157642 PMC2821115

[63] Conley TG Hansen CB Rossi PE. Plausibly exogenous. Rev Econ Stat. (2012) 94:260–72. 10.1162/REST_a_00139

[64] The United Nations. Available online at: https://www.un.org/sustainabledevelopment/ (accessed July 18, 2021).

[65] Chen Y Fanga H. The long-term consequence of China's “Later, Longer, Fewer” campaign in old age. J Dev Econ. (2021) 151:102664. 10.1016/j.jdeveco.2021.102664

[66] Yao Y Jiang L. Urbanization forces driving rural urban income disparity: evidence from metropolitan areas in China. J Clean Product. (2021) 312:127748. 10.1016/j.jclepro.2021.127748

[67] Zheng L Shepherd M Batuo ME. Variations in the determinants of regional development disparities in rural China. J Rural Stud. (2021) 82:29–36. 10.1016/j.jrurstud.2020.08.01128784132

[68] Zhu D Ye X Li W Ding R He P. Urban health advantage or urban health penalty? Urban-rural disparities in age trajectories of physiological health among Chinese middle-aged and older women and men. Health Place. (2021) 69:102559. 10.1016/j.healthplace.2021.102559 33773262

[69] *China, Women's News*. Available online at: http://news.cau.edu.cn/art/2019/2/4/art_8779_605868.html (accessed July 18, 2021).

[70] Budig MJ England P. The wage penalty for motherhood. Am Sociol Rev. (2001) 66:204–25. 10.2307/2657415

[71] Gangl M Ziefle A. Motherhood, labor force behavior, and women's career: an empirical assessment of the wage penalty for motherhood in Britain, German and the United States. Demography. (2009) 46:341–69. 10.1353/dem.0.0056 21305397 PMC2831275

[72] Jia N Dong X. Economic transition and the motherhood wage penalty in urban China: investigation using panel data. Cambridge J Econ. (2013) 37:819–43. 10.1093/cje/bes044

[73] Wang Q Lin M. Work-family policy and female entrepreneurship: evidence from China's subsidized child care program. China Econ Rev. (2019) 54:256–70. 10.1016/j.chieco.2018.11.008

[74] National Health Commission of the people's Republic of China. Available online at: http://www.nhc.gov.cn/wjw/tia/202007/7ca347a05ff143a7ac85d9ea1888f7f5.shtml (accessed July 18, 2021).

[75] Guo G. Maternal dilemma and gender anxiety in the discourse of widowed child-rearing. Soc Sci Beijing. (2019) 10:117–28. (in Chinese). 10.13262/j.bjsshkxy.bjshkx.191012

